# Association Between Clinical Practice Group Adherence to Quality Measures and Adverse Outcomes Among Adult Patients With Diabetes

**DOI:** 10.1001/jamanetworkopen.2019.9139

**Published:** 2019-08-14

**Authors:** Lauren G. Gilstrap, Michael E. Chernew, Christina A. Nguyen, Sartaj Alam, Barbara Bai, J. Michael McWilliams, Bruce E. Landon, Mary Beth Landrum

**Affiliations:** 1 The Dartmouth Institute, Dartmouth Medical School, Lebanon, New Hampshire; 2 Division of Cardiology, Dartmouth-Hitchcock Medical Center, Lebanon, New Hampshire; 3 Department of Health Care Policy, Harvard Medical School, Boston, Massachusetts; 4 Department of Medicine, Brigham and Women’s Hospital, Boston, Massachusetts; 5 Division of General Medicine, Beth Israel Deaconess Hospital, Boston, Massachusetts

## Abstract

**Importance:**

Clinical practice group performance on quality measures associated with chronic disease management has become central to reimbursement. Therefore, it is important to determine whether commonly used process and disease control measures for chronic conditions correlate with utilization-based outcomes, as they do in acute disease.

**Objective:**

To examine the associations among clinical practice group performance on diabetes quality measures, including process measures, disease control measures, and utilization-based outcomes.

**Design, Setting, and Participants:**

This retrospective, cross-sectional analysis examined commercial claims data from a national health insurance plan. A cohort of eligible beneficiaries with diabetes aged 18 to 65 years who were enrolled for at least 12 months from January 1, 2010, through December 31, 2014, was defined. Eligible beneficiaries were attributed to a clinical practice group based on the plurality of their primary care or endocrinology office visits. Data were analyzed from October 1, 2018, through April 30, 2019.

**Main Outcomes and Measures:**

For each clinical practice group, performance on current diabetes quality measures included 3 process measures (2 testing measures [hemoglobin A_1c_ {HbA_1c_} and low-density lipoprotein {LDL} testing] and 1 drug use measure [statin use]) and 2 disease control measures (HbA_1c_ <8% and LDL level <100 mg/dL). The rates of utilization-based outcomes, including hospitalization for diabetes and major adverse cardiovascular events (MACEs), were also measured.

**Results:**

In this cohort of 652 258 beneficiaries with diabetes from 886 clinical practice groups, 42.9% were aged 51 to 60 years, and 52.6% were men. Beneficiaries lived in areas that were predominantly white (68.1%). At the clinical practice group level, except for high correlation between the 2 testing measures, correlations among different quality measures were weak (*r* range, 0.010-0.244). Rate of HbA_1c_ of less than 8% had the strongest correlation with hospitalization for MACE (*r* = −0.046; *P* = .03) and diabetes (*r* = −0.109; *P* < .001). Rates of HbA_1c_ control at the clinical practice group level were not significantly associated with likelihood of hospitalization at the individual level. Performance on the process and disease control measures together explained 3.9% of the variation in the likelihood of hospitalization for a MACE or diabetes at the individual level.

**Conclusions and Relevance:**

In this study, performance on utilization-based measures—intended to reflect the quality of chronic disease management—was only weakly associated with direct measures of chronic disease management, namely, disease control measures. This correlation should be considered when determining the degree of financial emphasis to place on hospitalization rates as a measure of quality in treatment of chronic diseases.

## Key Points

**Question:**

What are the correlations between types of quality measures in treatment of diabetes, and how might these correlations be associated with quality-based reimbursement for chronic diseases?

**Findings:**

In this cross-sectional study of 652 258 adults with diabetes from 2010 to 2014 at the clinical practice group level, correlations among quality measures were weak, and process and disease control performance were not strongly associated with hospitalization rates. Process and disease control performance at the clinical practice group level explained 3.9% of the variation in hospitalization rates at the individual level.

**Meaning:**

These findings raise concern about the use of utilization-based outcomes (hospitalizations) as a measure of quality in chronic diseases.

## Teaser

This cross-sectional analysis examines the associations among clinical practice group performance on quality measures, including process measures, disease control measures, and utilization-based outcomes, among adults treated for diabetes.

## Introduction

Since the early 2000s, performance on quality measures has become central to the reimbursement of medical providers (defined as any health care professionals who directly care for patients and bill Medicare under their own license), most commonly within clinical practice groups and hospitals. Many commercial insurance companies now use pay-for-performance programs to define some percentage of provider reimbursement,^1^ and with the passage of the Medicare and CHIP Reauthorization Act of 2015, most providers now participate in a value-based payment program that relies on quality measurement to determine reimbursement.^2^ Therefore, how quality performance is measured and how those measures correlate with each other is of paramount importance to patients, providers, and payers.

Recently, payment models have gravitated away from traditional process and disease control measures and toward utilization-based outcomes, such as hospitalizations, to define and measure quality. This change is demonstrated most clearly by the evolution of the accountable care organization quality benchmark measures.^3,4^ Moreover, the Medicare Payment Advisory Commission recently recommended a focus on hospitalization rates as a population-based quality measure in alternative payment models^5^ and in their proposed replacement of the Merit-Based Incentive Payment System, largely to ease the burden of quality reporting.^6^

In acute disease, the associations between process measures, utilization-based outcomes, and patient outcomes (eg, mortality) are well established and have been previously studied.^7,8,9^ In chronic diseases, however, in addition to the challenges of time lags and lower mortality rates, the associations between different types of quality measures have not been well studied.^9^ Given these challenges and the increasing prevalence, cost, and complexity of chronic disease care, further work is timely to determine the association among traditional process measures of quality (eg, testing and drug use measures), which are easy to measure but may not be strongly correlated with outcomes^10,11^; disease control measures, which are costly to measure but have robust clinical validity^12^; and utilization-based outcomes, which are also easy to measure but of unclear validity in chronic diseases. This study examined the associations among quality metrics, including process, disease control, and utilization-based outcomes, for diabetes, one of the most common and clinically important chronic conditions,^13^ and explored the implications for quality-based reimbursement.

## Methods

### Study Population

We used medical and pharmacy claims from January 1, 2010, through December 31, 2014, and laboratory results from a large national health insurance plan. We obtained institutional review board approval from Harvard University’s Committee on the Use of Human Subjects, which waived the need for informed consent because the data sets were deidentified. This article is compliant with the Strengthening the Reporting of Observational Studies in Epidemiology (STROBE) reporting guideline for cross-sectional studies. All analyses were performed from October 1, 2018, through April 30, 2019.

Our sample included individuals aged 18 to 65 years who were enrolled continuously for at least 1 calendar (measurement) year from 2010 through 2014 with diabetes, defined by the 2013 Healthcare Effectiveness Data and Information Set criteria of more than 1 inpatient visit or more than 2 outpatient visits with an *International Classification of Diseases, Ninth Revision* (*ICD-9*) code for diabetes. Because pharmacy data were limited to beneficiaries with pharmacy benefits with the same insurer (48% for the diabetes cohort) and laboratory data were available for only some beneficiaries (52% for the diabetes cohort) depending on where they underwent laboratory testing, some quality measures were limited to these patient subsets.

### Patient Characteristics

Age and sex were obtained from the enrollment file. Other demographic and socioeconomic variables, including race/ethnicity (percentage white, black, or Hispanic), educational level (percentage with a college education), and median income, were determined using the beneficiaries’ zip codes and US Census data.^14^ Rural or urban residence was determined using the rural-urban commuting area code of the beneficiaries’ zip codes.^15^ Geographic region was determined using the beneficiaries’ zip codes and classified into 1 of 4 regions (Northeast, Midwest, South, and West) as defined by the Centers for Disease Control and Prevention.^16^ Type 1 diabetes was determined using *ICD-9* codes (250.x1 or 250.x3).^17^ Other comorbidities were measured during the calendar year using the DxCG intelligence tool’s proprietary algorithm.^18^

### Patient to Clinical Practice Group Attribution

Eligible patients were assigned to clinical practice groups, defined using tax identification numbers (TINs). A single TIN can represent a solo health care professional, a small physician group, or a larger group. Large organizations will occasionally bill using multiple TINs. Patients were assigned to clinical practice groups (TINs) using a modified version of the 2-step attribution rule of the Centers for Medicare & Medicaid Services.^19^ In each year, beneficiaries were assigned to a TIN using a plurality of primary care or, because we focus on patients with diabetes, endocrinology face-to-face office visits (weighted equally and identified using *Current Procedural Terminology* [*CPT*] codes 99201-99215, 99241-99245, G0402, G0438, and G0438), with specialty codes for family medicine (08), internal medicine (11), geriatric medicine (38), general provider organization (01), or endocrinology (46). If patients had the same number of visits to more than 1 TIN, they were assigned to the TIN with the greater sum of allowed costs. To ensure sufficient precision within a clinical practice group, we restricted our sample to clinical practice groups (TINs) with at least 40 attributed beneficiaries with diabetes, of whom 20 had to have laboratory and pharmacy data for every relevant measure.

### Process and Disease Control Measures

For each clinical practice group, we analyzed its rates of adherence to the diabetes quality measures most commonly used by current alternative payment models and quality measurement/reimbursement models (eTable 1 in the Supplement). These models included 3 process measures (2 testing-based measures [hemoglobin A_1c_ {HbA_1c_} and low-density lipoprotein {LDL} level] and 1 drug use measure [statin use]) and 2 disease control measures (HbA_1c_ <8% and LDL level <100 mg/dL; to convert HbA_1c_ to proportion of total hemoglobin, multiply by 0.01; LDL to millimoles per liter, multiply by 0.0259).^20,21,22^ Although the blood cholesterol guidelines switched from being LDL based to risk factor based in 2013,^23^ we included LDL level control because it is relevant for most of the time frame of our data (2010-2012 and most of 2013). The details of how each measure was coded are included in eTable 2 in the Supplement.

### Utilization-Based Outcome Rates

Next, we examined 2 commonly considered outcome measures: admissions for major adverse cardiovascular events (MACEs) and diabetes. Admissions for MACEs were determined using *ICD-9*, *CPT*, and Healthcare Common Procedure Coding System codes for acute coronary syndrome, stroke, malignant dysrhythmia, sudden cardiac death, and coronary revascularization (eTable 3 in the Supplement).^24,25,26^ Admissions for diabetes were determined using the Agency for Healthcare Research and Quality’s prevention quality indicator (PQI 93) for diabetes.^27^ The frequency distribution of indications for admission within PQI 93 is displayed in eTable 4 in the Supplement.

### Statistical Analysis

We first examined variation in the group-level rates of adherence to diabetes quality measures and admission rates and used hierarchical logistic regression models to compute risk-adjusted clinical practice group–level performance. Next, we examined the correlations among process measures, disease control measures, and utilization-based outcomes. Specifically, we computed mean rates for each measure in each group and pairwise correlations between the clinical practice groups. We then adjusted these correlations for all patient factors listed in Table 1. Because measurement error in group rates can bias estimated correlations toward zero, we adjusted the pairwise correlations using Spearman-Brown reliability adjustment to account for sampling variation in group-level estimates.^28,29^ This adjustment attenuates observed correlations by the estimated reliability of the measures that we compute as a function of the mean number of patients with diabetes within all physician practices and the intraclass correlation coefficients for each quality measure. We used a generalized estimating equation model with a logit link to determine the magnitude of association between clinical practice group performance on process and disease control measures and the likelihood of hospitalization for a MACE or diabetes at the individual level. This model was adjusted for patient-level variables. Two-sided *P* < .05 was considered significant in all analyses. All statistical analyses were performed using SAS version 9.4 (SAS Institute Inc) and R version 3.5.1 (R Project for Statistical Computing).

**Table 1.** Diabetes Cohort Baseline Characteristics, 2010-2014^a^

Characteristic	Data (N = 652 258)
Age group, y, No. (%)	
18-30	18 996 (2.9)
31-40	54 345 (8.3)
41-50	149 184 (22.9)
51-60	279 641 (42.9)
61-65	150 092 (23.0)
Sex, No. (%)	
Male	343 405 (52.6)
Female	308 853 (47.4)
Race/ethnicity, %^b^	
White	68
Black	17
Hispanic/Latino	18
Socioeconomic	
Median income, $/y	65 691.09
College educated, %^b^	29
Population, No. (%)	
Urban	614 068 (94.1)
Large rural	14 576 (2.2)
Small rural	3871 (0.6)
Isolated	3467 (0.5)
Unknown	16 276 (2.5)
Geography, No. (%)	
Northeast	157 274 (24.1)
Midwest	66 507 (10.2)
South	348 144 (53.4)
West	79 899 (12.2)
Unknown	434 (0.1)
Type 1 diabetes, No. (%)	
Yes	54 781 (8.4)
No	597 477 (91.6)
Comorbidities, No. (%)	
Hypertension	347 825 (53.3)
Hyperlipidemia	322 043 (49.4)
Atrial fibrillation	10 600 (1.6)
Chronic kidney disease	40 232 (6.2)
Chronic obstructive pulmonary disease	18 030 (2.8)
Heart failure	22 363 (3.4)
Ischemic heart disease/coronary artery disease	67 709 (10.4)

^a^ Defined as patients with diabetes and able to be attributed to a tax identification number with at least 40 patients with diabetes, in whom at least 20 have associated laboratory and pharmacy test data. Percentages have been rounded and may not total 100.

^b^ Number of patients are not shown because these are reported at the zip code level.

## Results

Baseline characteristics for the cohort with diabetes from 2010 through 2014 are displayed in Table 1. The diabetic cohort included 652 258 patients from 886 clinical practice groups. Most patients (42.9%) were aged 51 to 60 years, and slightly more were men than women (52.6% vs 47.4%). Most beneficiaries lived in areas that were predominantly white (68.1%), with a median area-level income of approximately $65 691.09 per year. Most (94.1%) resided in urban areas, and more than half (53.4%) lived in the South. Only 8.4% had type 1 diabetes. Approximately one-half had hypertension (53.3%) and hyperlipidemia (49.4%), but only 10.4% had ischemic heart disease.

Clinical practice group performance on current diabetes quality measures and their rates of hospitalization are displayed in Table 2. In terms of process measure performance, the mean (SD) rates of achievement were high for current testing measures of LDL level (83% [8%]; median, 84% [interquartile range {IQR}, 80%-88%]) and HbA_1c_ (87% [7%]; median, 88% [IQR, 84%-91%]), with little variation (coefficient of variation [CV], 9.3% and 7.5%, respectively) among clinical practice groups. Drug use rates were lower (mean [SD], 58% [9%]; median, 58% [IQR, 53%-63%]) but had more variation (CV, 15.0%). Disease control measures rates, including HbA_1c_ of less than 8% (mean [SD], 70% [8%]; median, 70% [IQR, 65%-75%]; CV, 12.1%) and LDL levels of less than 100 mg/dL (mean [SD], 59% [8%]; median, 59% [IQR, 54%-64%]; CV, 13.6%), were slightly higher than drug use rates and had similar variation among clinical practice groups. The rates of hospitalization for MACEs (mean [SD], 2% [1%]; median, 1% [IQR, 1%-2%]; CV, 78.6%) and diabetes (mean [SD], 3% [3%]; median, 2% [IQR, 1%-3%]; CV, 66.8%) were low in this population but demonstrated high rates of variation among clinical practice groups.

**Table 2.** Clinical Practice Group Performance on Current Process and Disease Control Measures and Rates of Utilization-Based Outcomes

Measure	Mean (SD) Performance, %			2010-2014 Performance, Median (IQR) [Difference], %	2010-2014 Coefficient of Variation, %
	2010	2014	2010-2014		
Testing					
HbA_1c_ test	87 (5)	87 (8)	87 (7)	88 (84-91) [7]	7.5
LDL test	84 (7)	82 (9)	83 (8)	84 (80-88) [8]	9.3
Drug use					
Statin use	58 (9)	60 (8)	58 (9)	58 (53-63) [10]	15.0
Disease control					
HbA_1c_ <8%	70 (9)	66 (8)	70 (8)	70 (65-75) [10]	12.1
LDL level <100 mg/dL	60 (8)	58 (8)	59 (8)	59 (54-64) [10]	13.6
Utilization-based outcome					
MACE hospitalization^a^	2 (1)	1 (1)	2 (1)	1 (1-2) [1]	78.6
Diabetes hospitalization	2 (2)	3 (2)	3 (3)	2 (1-3) [2]	66.8

Abbreviations: HbA_1c_, hemoglobin A_1c_; IQR, interquartile range; LDL, low-density lipoprotein; MACE, major adverse cardiovascular event.

SI conversion factors: to convert HbA_1c_ to proportion of total hemoglobin, multiply by 0.01; LDL to millimoles per liter, multiply by 0.0259.

^a^ Includes admission for acute coronary syndrome, stroke, malignant dysrhythmia, sudden cardiac death, and coronary revascularization.

Correlations between different quality measures at the group level were highly variable; when adjusted for patient covariates, they became closer to zero. Unadjusted correlations are displayed in eTable 5 in the Supplement. Adjusted correlations are displayed in Table 3. Hemoglobin A_1c_ of less than 8% had the strongest correlation with hospitalization for MACEs (*r* = −0.046; *P* = .03), whereas LDL testing had the weakest correlation (*r* = −0.013; *P* > .05). Hemoglobin A_1c_ of less than 8% also had the strongest correlation with hospitalization for diabetes (*r* = −0.109; *P* < .001), whereas LDL levels of less than 100 mg/dL had the weakest (*r* = −0.047; *P* = .02).

**Table 3.** Adjusted Correlations Among Performance on Diabetes Process, Disease Control Quality Measures, and Utilization-Based Outcomes^a^

Measure	Correlation, *r *value				
	Testing Measures		Drug Use Measure, Any Statin	Disease Control Measures	
	HbA_1c_ Test	LDL Test		HbA_1c_ <8%	LDL Level <100 mg/dL
Testing					
HbA_1c_ test	NA	NA	NA	NA	NA
LDL test	0.812^b^	NA	NA	NA	NA
Drug use					
Any statin	NA	0.116^b^	NA	NA	NA
Disease control					
HbA_1c_ <8%	0.010	0.016	0.049^c^	NA	NA
LDL level <100 mg/dL	−0.015	−0.016	0.244^b^	0.130^b^	NA
Utilization-based outcome					
MACE hospitalization^d^	−0.022	−0.013	0.043^c^	−0.046^c^	−0.041^c^
Diabetes hospitalization	−0.054^e^	−0.090^b^	−0.077^b^	−0.109^b^	−0.047^c^

Abbreviations: HbA_1c_, hemoglobin A_1c_; LDL, low-density lipoprotein; MACE, major adverse cardiovascular event; NA, not applicable.

SI conversion factors: to convert HbA_1c_ to proportion of total hemoglobin, multiply by 0.01; LDL to millimoles per liter, multiply by 0.0259.

^a^ All correlations are shown at the clinical practice group level and adjusted for year and patient-level variables, including age, sex, race, ethnicity, college education, median income, population density, geographic region, type 1 diabetes, hypertension, hyperlipidemia, atrial fibrillation, chronic kidney disease, chronic obstructive pulmonary disease, heart failure, and ischemic heart disease.

^b^ *P* < .001.

^c^ *P* < .05.

^d^ Includes admission for acute coronary syndrome, stroke, malignant dysrhythmia, sudden cardiac death, and coronary revascularization.

^e^ *P* < .01.

Next, we examined the association among clinical practice group performance on process and disease control measures and utilization-based outcomes at the enrollee level. The results are displayed in Table 4. Three quality measures were significantly associated with hospitalizations, including LDL testing (estimate, −1.223 [95% CI, −1.950 to −0.497]), statin use (estimate, −0.603 [95% CI, −0.985 to −0.222]), and LDL level of less than 100 mg/dL (estimate, −0.364 [95% CI, −0.703 to −0.026]). A 10–percentage point improvement in statin use at the clinical group level was associated with an 11% decrease in the odds of hospitalization (odds ratio [OR], 0.89; 95% CI, 0.82-0.95), and a 10–percentage point improvement in the rates of LDL levels of less than 100 mg/dL was associated with a 6% decrease in odds of hospitalization (OR, 0.94; 95% CI, 0.91-0.98). Rates of HbA_1c_ of less than 8% at the clinical practice group level were not significantly associated with an individual’s likelihood of hospitalization. Together, performance on these 5 measures of quality explained 3.9% of the variation in likelihood of hospitalization for a MACE or diabetes at the individual level.

**Table 4.** Estimate of Clinical Practice Group Quality Performance on Hospitalization Rates for Major Adverse Cardiovascular Events or Diabetes^a^

Measure	Estimate (95% CI)	*P* Value^b^
Testing		
HbA_1c_ test	0.104 (−0.757 to 0.965)	.81
LDL test	−1.223 (−1.950 to −0.497)	.001
Drug use		
Statin use	−0.603 (−0.985 to −0.222)	.002
Disease control		
HbA_1c_ <8%	−0.274 (−0.691 to 0.142)	.20
LDL level <100 mg/dL	−0.364 (−0.703 to −0.026)	.04

Abbreviations: HbA_1c_, hemoglobin A_1c_; LDL, low-density lipoprotein; MACE, major adverse cardiovascular event.

SI conversion factors: to convert HbA_1c_ to proportion of total hemoglobin, multiply by 0.01; LDL to millimoles per liter, multiply by 0.0259.

^a^ Analysis performed at the clinical practice group level and adjusted for year and patient-level variables, including age, sex, race, ethnicity, college education, median income, population density, geographic region, type 1 diabetes, hypertension, hyperlipidemia, atrial fibrillation, chronic kidney disease, chronic obstructive pulmonary disease, heart failure, and ischemic heart disease.

^b^ Calculated using the generalized estimating equation model.

## Discussion

We analyzed a large, national cohort of commercially insured patients aged 18 to 65 years to determine the associations among process (testing and drug use) measures, disease control measures, and utilization-based outcomes in diabetes, a common chronic condition. We found testing measure rates to be high with little variation among clinical practice groups, whereas drug use and disease control measure rates were lower with more variation between clinical practice groups. The rates of hospitalization were low but demonstrated greater variability among clinical practice groups. In addition, we found that associations between process, disease control, and utilization-based outcomes were low and became even smaller when adjusted for patient covariates. Finally, we found that changes in a clinical practice group’s performance on current process and disease control measures had a minimal association with its rates of hospitalization for MACEs or diabetes and that overall clinical practice group performance on those 5 measures explained only 3 clinical practice group rates of hospitalization (LDL testing, statin use, and LDL level <100 mg/dL), which were significantly associated with hospitalization at the individual level.

The correlations described in this study were weaker than those previously described in acute diseases. In a population of patients hospitalized with acute myocardial infarction, the correlation between process measure performance and patient outcomes was higher (*r* = 0.40; *P* < .001).^7,8^ This stronger correlation may be due to the sicker nature of hospitalized patients or to the lack of a time lag, higher event rates, and less confounding in acute diseases. By comparison, in our study, the strongest correlation we observed between process or disease control measures and utilization-based outcomes was only −0.109. Given that the observed correlations decreased with adjustment for patient covariates, we can also conclude that the negative correlations between process or disease control measuews and utilization-based measures were weak and likely biased away from the null by residual confounding. Thus, the true correlation, without selection effects, would be even weaker or perhaps even positive. We believe that this outcome is a significant absence of a correlation that has been widely presumed to be present in prior iterations of quality measurement in chronic diseases. To further substantiate this finding, we estimated the association of changes in a clinical practice group’s quality measure performance on hospitalization rates. We found all of these estimates to be quite small. We determined that a clinical practice group’s performance on these 5 measures taken together explained only 3.9% of the variation between groups’ rates of hospitalizations.

These findings have several potential explanations. First, as demonstrated in this study, process measures are topped out, with little variation among clinical practice groups. Although not surprising in light of the incentives that have been placed on process measures in recent decades,^30^ these measures explain their limited ability to discriminate among clinical practice groups. In contrast, drug use and disease control measures demonstrated lower levels of achievement and higher levels of variation, suggesting that these measures may be better able to distinguish quality among clinical practice groups. Whether it is practically feasible, however, to calculate drug use and disease control measures for large populations remains unknown.

A potential explanation for the weak association among drug use, disease control performance, and utilization-based outcome rates is that higher hospitalization rates may not always be indicative of poor outpatient care in chronic diseases, such as diabetes. To test this hypothesis, we estimated the association of changes in a clinical practice groups’ quality measure achievement rate on their patients’ individual rates of hospitalization for a MACE or diabetes. Perhaps most interestingly, rates of change in HbA_1c_ of less than 8% were not significantly associated with hospitalization rates at the clinical practice group level. Because we know from robust clinical trial data that improved disease control results in better outcomes at the individual patient level, these findings at the clinical group level suggest that, on the margin, higher hospitalization rates may not always be indicative of poorer outpatient care and that the use of hospitalization rates as a measure of clinical quality in chronic diseases may merit additional study.

Moreover, in contrast to prior work in acute diseases in which quality performance on process and disease control measures explained approximately 6% of the variation in patient outcomes,^7^ in this study, clinical practice group performance on all 5 process and disease control measures explained only 3.9% of the variation in utilization-based outcome rates between clinical practice groups. This finding suggests, at the very least, that clinical practice groups’ performance on current quality measures should not be used to predict utilization-based outcome rates in chronic diseases and begins to question the validity of using utilization-based outcome rates as a quality measure in chronic diseases.

### Limitations

This study does not attempt to prove (or disprove) a causal relationship between quality measure performance and utilization-based outcome rates. Rather, this study describes the associations between quality performance on commonly used quality metrics at the clinical practice group level. To that end, this study uses administrative data that lack the granularity of clinical data, and we are limited in our ability to adjust for only patient-level characteristics that are identifiable in claims data. Although all of the correlations and models presented are adjusted for patient-level characteristics, adjustment for unmeasured confounding would likely result in lower correlations at the clinical practice group level. Although these lower correlations may limit the predictive capacity of our models, they accurately mirror the real world, where payers often only have access to administrative claims data. Second, this work, like current reimbursement methods, relies on accurately attributing patients to a provider and/or a clinical practice group responsible for most of their care.^31^ The current Centers for Medicare & Medicaid Services algorithm attributes patients to a primary care clinical practice group; this study did the same and added endocrinologists to reflect the study’s focus on diabetes care. Notably, allowing endocrinology visits into the attribution algorithm could bias the associations. For example, if patients with worse diabetes see their endocrinologist more often or if they see an endocrinologist more often after hospitalizations or when HbA_1c_ increases, that factor could strengthen the observed association between HbA_1c_ of less than 8% and hospitalizations for diabetes. However, although the association between HbA_1c_ level and admission for diabetes was the strongest association we observed between utilization-based outcomes and quality measure performance, it remains weak by traditional standards. Third, we consider LDL levels of less than 100 mg/dL as a disease control measure. Although this definition does not reflect the 2013 Cholesterol Guideline change (which eliminated specific LDL targets),^32^ it is appropriate for most of this clinical period and still reflects much of clinical practice.^33^ In addition, these results were derived from the commercially insured population aged 18 to 65 years. Associations may be higher (or lower) among patients who are more likely to experience frequent hospitalizations (ie, older patients and those with less socioeconomic support). This study is cross-sectional and does not attempt to provide a causal relationship between disease control performance and utilization-based outcomes at an individual level. Rather, this study seeks to compare quality performance and clinical outcomes by a clinical practice group in the same year in the attempt to better understand the implications of using these metrics as measures of quality in chronic diseases.

## Conclusions

In this study, the associations among different types of diabetes quality measures were weak, and much variation in the rates of utilization-based outcomes was unexplained by clinical practice group performance on traditional process and disease control measures. This outcome may be due in part to the topped-out nature of process measures, but the weak association between clinically robust disease control measures and hospitalization rates, the modest difference in hospitalization rates based on process and disease control performance, and the small amount of variation between clinical practice group hospitalization rates explained by process and disease control performance all raise concern about the validity of utilization-based outcomes as a measure of quality in chronic diseases. In chronic diseases such as diabetes, more hospitalizations may not necessarily be evidence of poor outpatient care, which has significant implications for quality-based reimbursement in chronic disease management.

## Notes

Supplement. **eTable 1.** Quality Metrics for Diabetes **eTable 2.** Quality Metric Coding **eTable 3.** ICD9/CPT/HCPCS Codes Used to Identify Major Adverse Cardiovascular Events (MACE) **eTable 4.** Distribution of PQI 93 Indications for Hospitalization **eTable 5.** Unadjusted Correlations Between Performance on Diabetes Quality Measures
