# Supplementary material for: Association Between Clinical Practice Group Adherence to Quality Measures and Adverse Outcomes Among Adult Patients With Diabetes
Source: JAMA Netw Open. 2019 Aug 14;2(8):e199139. doi: 10.1001/jamanetworkopen.2019.9139 (PMC6694385; doi:10.1001/jamanetworkopen.2019.9139)
Supplement: Supplement. — eTable 1. Quality Metrics for Diabetes eTable 2. Quality Metric Coding eTable 3. ICD9/CPT/HCPCS Codes Used to Identify Major Adverse Cardiovascular Events (MACE) eTable 4. Distribution of PQI 93 Indications for Hospitalization eTable 5. Unadjusted Correlations Between Performance on Diabetes Quality Measures [file jamanetwopen-2-e199139-s001.pdf]

Supplementary Online Content

Gilstrap LG, Chernew ME, Nguyen CA, et al. Association between clinical practice group adherence to quality measures and adverse outcomes among adult patients with diabetes. *JAMA Netw Open.* 2019;2(8):e199139. doi:10.1001/jamanetworkopen.2019.9139

eTable 1. Quality Metrics for Diabetes

eTable 2. Quality Metric Coding

eTable 3. ICD9/CPT/HCPCS Codes Used to Identify Major Adverse Cardiovascular Events (MACE)

eTable 4. Distribution of PQI 93 Indications for Hospitalization

eTable 5. Unadjusted Correlations Between Performance on Diabetes Quality Measures

This supplementary material has been provided by the authors to give readers additional information about their work.

© 2019 Gilstrap LG et al. *JAMA Network Open.*

eTable 1. Quality Metrics for Diabetes

HEDIS 2013	MACRA/MIPS	Consensus Core Set
Glycemic Control		
HbA1c testing	HbA1c control	HbA1c testing
HbA1c control		HbA1c control
Cardiovascular Risk Factor Management		
*Blood Pressure good control (<140/80mmHg)		
*Blood Pressure good control (<140/90mmHg)		
LDL testing		
LDL control		
	Statin therapy	
Microvascular Disease Screening		
*Eye exam	*Eye exam	*Eye Exam
*Medical Attention for Nephropathy	*Medical Attention for Nephropathy	*Medical Attention for Nephropathy
	*Foot Exam	*Foot Exam
	*Diabetic retinopathy – communication with the physician managing ongoing diabetes care	
	*Diabetic foot and ankle care, ulcer prevention – evaluation of footwear	
	*Diabetic foot and ankle care, peripheral neuropathy – neurological evaluation	

* Indicates that this metric was excluded from these analyses. Blood pressure metrics were excluded because we did not have the clinical information (recorded blood pressures) necessary to determine rates of adherence to this metric. Eye exam and foot exam (including “diabetic foot and ankle care, peripheral neuropathy - neurological evaluation”) were excluded because we were unable to reliably capture these exams when performed by primary care physicians as part of an annual exam. “Medical attention for nephropathy” was excluded due to inconsistent and incomplete use of CPT II use in our dataset. “Diabetic retinopathy - communication with the physician managing ongoing diabetes care” could not be reliably captured in our data. “Diabetic foot and ankle care – ulcer prevention” could not be assessed because we were not able to determine when evaluation for diabetic footwear had occurred.

© 2019 Gilstrap LG et al. *JAMA Network Open.*

eTable 2. Quality Metric Coding

Process Measures (which include both "testing" and "drug-use" measures)

Testing Measures

Metric	Numerator	Denominator
**% with HbA1c Test**	Eligible beneficiaries with ≥1 laboratory test for HbAlc (from the laboratory file) during the measurement year	All eligible beneficiaries*
**% with LDL Test**	Eligible beneficiaries with ≥1 laboratory test for LDL (from the laboratory file) during the measurement year	All eligible beneficiaries*

** Eligible Beneficiaries: beneficiates with a diagnosis of diabetes (≥1 inpatient or ≥2 outpatient claims for diabetes during the measurement year), laboratory and pharmacy data attributed to a TIN with ≥ 20 attributed beneficiaries.*

Drug-Use Measure

Metric	Numerator	Denominator
**% with use of any statin**	Eligible beneficiaries* with ≥1 fill of any statin. Statin use identified in the pharmacy file using National Drug Codes (NDC) † during the measurement year	All eligible beneficiaries*

** Eligible Beneficiaries: beneficiates with a diagnosis of diabetes (≥1 inpatient or ≥2 outpatient claims for diabetes during the measurement year), laboratory and pharmacy data attributed to a TIN with ≥ 20 attributed beneficiaries.*

† Statin NDC codes obtained from HEDIS 2016 and are available at: http://www.ncqa.org/hedis-quality-measurement/hedis-measures/hedis-2016/hedis-2016-ndc-license/hedis-2016-final-ndc-lists (Access Date March 21, 2019).

© 2019 Gilstrap LG et al. *JAMA Network Open.*

<u>Disease Control Measures</u>

**% with HbA1c <8%**	Eligible beneficiaries* whose first HbA1c value in the measurement year was ≥8%	Eligible beneficiaries* with ≥1 laboratory test for HbAlc (from the laboratory file) during the measurement year
**% with LDL <100mg/dl**	Eligible beneficiaries* whose first LDL value in the measurement year was ≥100mg/dl	Eligible beneficiaries* with ≥1 laboratory test for LDL (from the laboratory file) during the measurement year

** Eligible Beneficiaries: beneficiates with a diagnosis of diabetes (≥1 inpatient or ≥2 outpatient claims for diabetes during the measurement year), laboratory and pharmacy data attributed to a TIN with ≥ 20 attributed beneficiaries.*

© 2019 Gilstrap LG et al. *JAMA Network Open.*

eTable 3. ICD9/CPT/HCPCS Codes Used to Identify Major Adverse Cardiovascular Events (MACE)

Acute Coronary Syndrome: 410.x

Angina: 411.1, 411.8x, 413.x

Cerebrovascular accident/stroke: 430-432, 433-436

Malignant dysrhythmia: 427.1, 427.4, 427.41-427.42, 427.5

Sudden Cardiac Death: 798.1, 798.2

Coronary Revascularization CPT: 33510-33519, 33520-33523, 33530-33536, 92973-92984, 92995-92998

Coronary Revascularization HCPCS: S2205-S2209, G0290, G0291

© 2019 Gilstrap LG et al. *JAMA Network Open.*

eTable 4. Distribution of PQI 93 Indications for Hospitalization

Index Year	PQI 01 Diabetes Short-term Complications Admission Rate	PQI 03 Diabetes Long-term Complications Admission Rate	PQI 14 Uncontrolled Diabetes Admission Rate	PQI 16 Lower-Extremity Amputation among Patients with Diabetes Rate	PQI93 Diabetes Admissions Composite
**2010**	637	1856	1164	49	3422
**2011**	660	1701	1180	55	3321
**2012**	623	1840	1207	71	3425
**2013**	658	1559	1066	50	3045
**2014**	801	1904	1171	71	3597

© 2019 Gilstrap LG et al. *JAMA Network Open.*

eTable 5. Unadjusted Correlations between Performance on Diabetes Quality Measures

	Testing Measures		Drug Use Measure	Disease Control Measures	
	**% with Hb1c Test**	**% with LDL Test**	**% Use of Any Statin**	**% HbAlc <8%**	**% LDL <100**
**Testing Measures**					
% with Hb1c Test					
% with LDL Test	0.836***				
**Drug-Use Measures**					
% Use of Any Statin	0.288***	0.325***			
**Disease Control Measures**					
% HbAlc <8%	0.182***	0.205***	0.213***		
% LDL <100mg/dl	0.116***	0.093***	0.305***	0.240***	
**Utilization-Based Outcomes**					
% with MACE Admission	-0.094***	-0.064***	-0.002***	-0.062***	-0.055***
% with Diabetes Admission	-0.113***	-0.162***	-0.147***	-0.170***	-0.087***

All correlations are shown at the provider group level. **p<0.05, **p<0.01, ***p<0.001.*

MACE is major adverse cardiovascular event and includes admission for acute coronary syndrome, stroke, malignant dysrhythmia, sudden cardiac death and coronary revascularization

© 2019 Gilstrap LG et al. *JAMA Network Open.*
